# Beyond biological risk: socioeconomic vulnerability and adverse pediatric kidney transplant outcomes

**DOI:** 10.1007/s00467-026-07322-6

**Published:** 2026-05-13

**Authors:** Sarah Kizilbash, Chung-II Wi, Sandra Amaral, Madison Beenken, David Watson, Warren McKinney, Young Juhn

**Affiliations:** 1 Department of Pediatrics, University of Minnesota, Minneapolis, USA; 2 Precision Population Science Lab and Department of Pediatric and Adolescent Medicine, Mayo Clinic, Rochester, MN, USA; 3 Dept of Pediatrics and Epidemiology, Children’s Hospital of Phila, Univ Pennsylvania Perelman School of Medicine, Philadelphia, PA, USA; 4 Precision Population Sciency Lab, Department of Quantitative Health Science, Mayo Clinic, Rochester, MN, USA; 5 Division of Nephrology, Hennepin Healthcare Research Institute, Minneapolis, MN, USA; 6 Department of Medicine, Hennepin Healthcare, Minneapolis, MN, USA; 7 Precision Population Science Lab and Department of Pediatric and Adolescent Medicine/Internal Medicine, Mayo Clinic Rochester, Mayo Clinic Health System, Rochester, MN, USA

**Keywords:** Socioeconomic status, Pediatric kidney transplant, Social determinants of health, Graft survival, Healthcare disparities

## Abstract

**Background:**

Adverse social determinants are known contributors to poorer long-term allograft outcomes. Yet optimal measures of social determinants are lacking. We sought to evaluate the association between socioeconomic status (SES) and graft loss in children using several different metrics of social determinants of health (SDOH).

**Methods:**

Our study included 137 pediatric kidney transplant recipients (< 18 years at kidney failure onset) transplanted between 2010 and 2020. The association between SES measures (individual- and area-level) and graft survival was examined using Cox regression models. Measures were dichotomized: HOUSES Index [low SES(Q1) vs. high (Q2–Q4)], Area Deprivation Index (ADI) [high deprivation (76–100 percentile) vs. low (1–75 percentile)], and Childhood Opportunity Index (COI) [low opportunity (1s–25 percentile) vs. high (26–100)]. All models were adjusted for transplant age, sex, donor type, pretransplant dialysis, insurance, kidney failure cause, and body mass index *z*-score.

**Results:**

After adjusting for covariates, we observed a significantly increased risk of graft loss in HOUSES Q1 vs. Q2–4 recipients (adjusted hazard ratio [aHR]: 3.25; 95% CI: 1.38, 7.64; *p* = 0.007) and nationally standardized low-opportunity COI vs. high-opportunity COI recipients (aHR: 5.01; 95% CI: 2.01, 12.5; *p* = 0.001). In contrast, we observed no significant association between ADI and graft loss.

**Conclusions:**

Using the HOUSES Index and COI, we identified a 3-fold to fivefold higher risk of pediatric graft loss among recipients with lower SDOH. These tools provide robust non-biological predictors for transplant outcomes; however, larger studies are required to compare their relative impact.

## Introduction

Kidney transplantation is the treatment of choice for kidney failure in children, affording a survival benefit that extends beyond 25 years compared to dialysis [1]. Kidney transplantation also offers improved growth, superior neurocognitive outcomes, and a better overall quality of life [2]. However, graft survival is not indefinite. Despite significant improvements in short-term graft survival, proportionate gains in long-term graft survival remain elusive. The 10-year graft failure rates are 19.7% for living-donor and 35.4% for deceased-donor pediatric kidney transplant recipients [3]. In 2023, 17.2% of the pediatric candidates on the United States (US) waitlist were awaiting retransplantation [3], further illustrating the burden of graft loss in this population and underscoring the importance of identifying biological and non-biological risk factors to protect graft longevity.

Social drivers of health (SDOH) are non-biological risk factors associated with a wide array of adverse health outcomes. Studies indicate that SDOH, such as language barriers, socioeconomic status (SES), and racial/ethnic minority status, are associated with reduced access to living-donor and preemptive transplants [4–6]. Similarly, racial/ethnic minority status is associated with adverse transplant outcomes; notably, Black children experience nearly double the graft failure rates compared to White children [7, 8]. Race and SES are intricately intertwined in the US, with poverty rates significantly higher among Black, Hispanic, and Native Americans compared to White individuals [9]. The link between race and SES raises the question of the extent to which the association between race and adverse transplant outcomes is mediated by SES. While race and ethnicity are not modifiable, SES, defined as one’s capacity to access and utilize desired resources [10, 11], can potentially be modified with targeted support and interventions [12]. Therefore, a granular investigation into the multifaceted effects of SES on graft outcomes is essential to inform targeted interventions aimed at mitigating non-biological threats to graft survival.

Pediatric studies examining the association between SES and kidney transplant outcomes remain scarce. Most existing studies define SES using zip code or census tract data [13, 14], an approach that may misclassify individual-level SES in up to 43% of instances [15, 16]. While area-level indices may be imprecise proxies for individual-level SES [16–19], evaluating both measures is essential as they offer complementary yet distinct insights into the various mechanisms driving disparities. Therefore, the objective of this study was to evaluate the effects of individual- and area-level SES on kidney graft survival in pediatric kidney transplant recipients. We hypothesized that lower SES would be associated with lower graft survival, and that individual-level SES would offer distinct insights compared to area-level measures.

## Method

### Study design

This retrospective cohort study was approved by the institutional review board (IRB) at the University of Minnesota (STUDY00009897). The clinical and research activities reported are consistent with the Principles of the Declaration of Istanbul as outlined in the ‘Declaration of Istanbul on Organ Trafficking and Transplant Tourism’.

### Study population

Using a prospectively maintained, IRB-approved, solid organ transplant database at the University of Minnesota, we identified all pediatric kidney transplant recipients (age < 18 years at onset of kidney failure) who were transplanted at the University of Minnesota between 1/1/2010 and 1/1/2020 and were residents of Minnesota at the time of their transplant. The transplant database contains post-transplant, peri-transplant, and pretransplant data on recipients and donors. Information on demographics, pretransplant comorbidities, transplant surgery variables, post-transplant complications, and outcomes is captured via manual chart abstraction and electronic data transfer from the electronic medical record. The study cohort was restricted to Minnesota residents, as the HOUSES Index was only available for Minnesota when the study was undertaken.

Recipients transplanted before age 2 were excluded to improve the generalizability of our findings, as most transplant centers do not perform kidney transplants in children below this age. Furthermore, restricting the cohort to children aged 2 years and older allowed for consistent BMI z-score computation across all recipients. For patients with multiple transplants during the study period, the first kidney transplant was included.

### Study variables

Our study included the following variables: age at kidney failure, age at transplant, cause of kidney failure, sex, race, donor source, pretransplant dialysis, prior transplant, insurance type, recipient’s body mass index (BMI) at transplant, and cause of graft loss.

Our exposures of interest included an individual-level SES measure, HOUSES, and area-level SES measures, namely ADI and COI.

### HOUSES

HOUSES is a robust, individual-level SES measure derived from four property variables: number of bedrooms, number of bathrooms, square footage, and estimated building value. These data are obtained from the County Assessor’s office and normalized within each county. To calculate the index, each property item is standardized into a z-score and then aggregated into a composite score; a higher HOUSES value reflects a higher SES. The HOUSES Index is divided into quartiles (Q1 indicating the lowest SES). HOUSES has been extensively validated and reported in 41 publications (see Supplementary Tables 1 and 2), showing its associations with 62 outcomes in approximately 1.5 million patients. To make HOUSES scalable, an automated cloud-based platform was recently established, making the HOUSES formulation process automatic and making HOUSES available to nationwide users. The HOUSES database is updated annually as property information is re-appraised. For this study, we retrieved recipient addresses at the time of transplant from the University of Minnesota transplant registry and computed HOUSES quartile for each recipient using the HOUSES Index computing algorithm. The HOUSES quartiles were dichotomized into low SES (Q1) and high SES (Q2–4). This approach was consistent with our previous studies evaluating the association between HOUSES and graft survival in adult kidney transplant recipients [19, 20].

### COI

The COI (3.0–2023) is a composite measure of neighborhood opportunities for children based on data from 2012 to 2023 for every neighborhood across the United States. It includes 44 indicators across three domains—education, health and environment, and social and economic—and 14 subdomains, including early childhood education, elementary education, secondary and post-secondary education, education resources, pollution, healthy environments, safety, and health-related resources, economic opportunities, economic resources, concentrated inequity, housing resources, social resources, and wealth [21]. Although originally developed at the census tract level, the census tract COI was subsequently matched to ZIP codes. We used ZIP code-level COI standardized at the national (COI_NAT_) and state ( COI_ST_) levels for assessment of neighborhood deprivation at the time of transplant. For analysis, the index was dichotomized into low-opportunity (1st–25th percentiles) and high-opportunity (26th–100th percentiles), hereafter referred to as low COI_NAT/ST_ and high COI_NAT/ST_.

### ADI

The ADI is a validated measure of SDOH based on 17 indicators related to education, employment, housing quality, and poverty. It was originally derived from US Census data and later updated with 2013 American Community Survey data, and it is determined at the census block group [22]. The ADI is reported in national percentiles, and for this comparison, was dichotomized into high-deprivation ADI (percentiles 76–100, or low SES) and low-deprivation ADI (percentiles 1–75, or high SES).

### Study outcome

Our primary outcome of interest was death-censored graft survival. Graft loss was defined as a return to dialysis or retransplantation. Recipients were followed from the date of transplant to the earliest of the date of graft loss, the date of death, or the last known date alive. The follow-up was censored at death or the end of follow-up.

### Statistical analysis

Descriptive statistics were used to summarize the characteristics of the cohort. We used Analysis of Variance (ANOVA) and t-tests for continuous variables and Pearson *χ*^2^ and Fisher exact tests for categorical variables to measure the association between SES measures and patient and transplant characteristics. We used Cox proportional hazards regression models to evaluate the association of SES measures with graft survival. In the model for HOUSES, a random effect (i.e., frailty) term for the county was used because the HOUSES Index is standardized within each county. The covariates included in the models were selected a priori based on their known association with the outcome of interest, namely age at transplant, sex (male or female), pretransplant dialysis (yes or no), donor type (living or deceased), insurance (public or private), cause of kidney failure (congenital anomalies, focal segmental glomerulosclerosis, glomerulonephritis, other, and unknown), and BMI (as standardized *z*-score) [23–25]. Adjusted Kaplan–Meier curves were used to show differences in graft survival between low SES and high SES groups [26]. All analyses were performed using R [27].

## Results

### Patient and transplant characteristics

Of the 151 recipients with complete data, 1 was excluded for missing neighborhood deprivation index (ADI), and 13 were excluded for being younger than 2 years of age at the time of transplant. Our final study cohort included 137 pediatric kidney transplant recipients; the median age at transplant was 12.1 (range: 2.06, 19.6) years, 46.0% were female, and 75.2% were White. Of the recipients, 54.7% received a living donor transplant, 29.2% received a preemptive transplant, and 60.6% had private insurance. The median BMI *z*-score of the cohort was 0.069 (range: −4.1, 2.4).

Table 1 presents patient characteristics by HOUSES Index. Compared to Q2–4 recipients, Q1 recipients were less likely to receive a preemptive transplant (*p* = 0.01) and more likely to have a prolonged pretransplant dialysis exposure (*p* = 0.01). We observed no differences in donor type and cause of kidney failure by HOUSES Index.

Table 2 shows patient characteristics by COI_NAT_. Children in the low COI_NAT_ group were more likely to be on public insurance (*p* = 0.006), more likely to have prolonged pretransplant dialysis exposure (*p* = 0.001), and more likely to receive a deceased donor transplant (*p* = 0.03). We also observed significant differences in the cause of kidney failure between the low COI_NAT_ and high COI_NAT_ groups (*p* = 0.039).

Patient characteristics by ADI and COI_ST_ are given in Supplementary Tables 3 and 4, respectively.

### HOUSES and graft survival

**Fig. 1:**
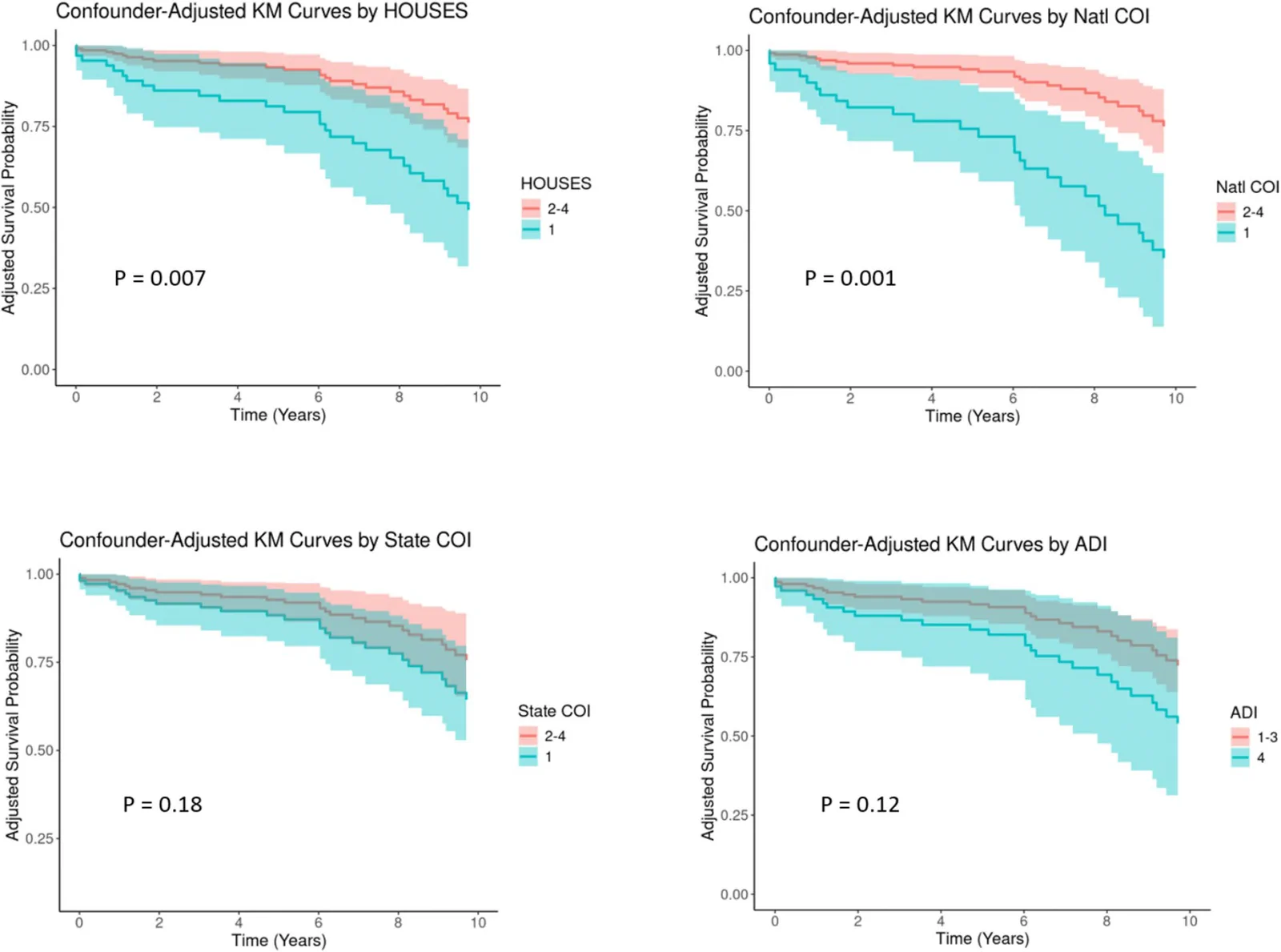
Graft survival by SES

We observed no significant association between HOUSES and delayed graft function (Q1 vs. Q2–4: 5.3% vs. 3.0%; *p* = 0.53). Figure 1 compares confounder-adjusted graft-survival Kaplan–Meier curves between Q1 and Q2–4 recipients.

**Fig. 2:**
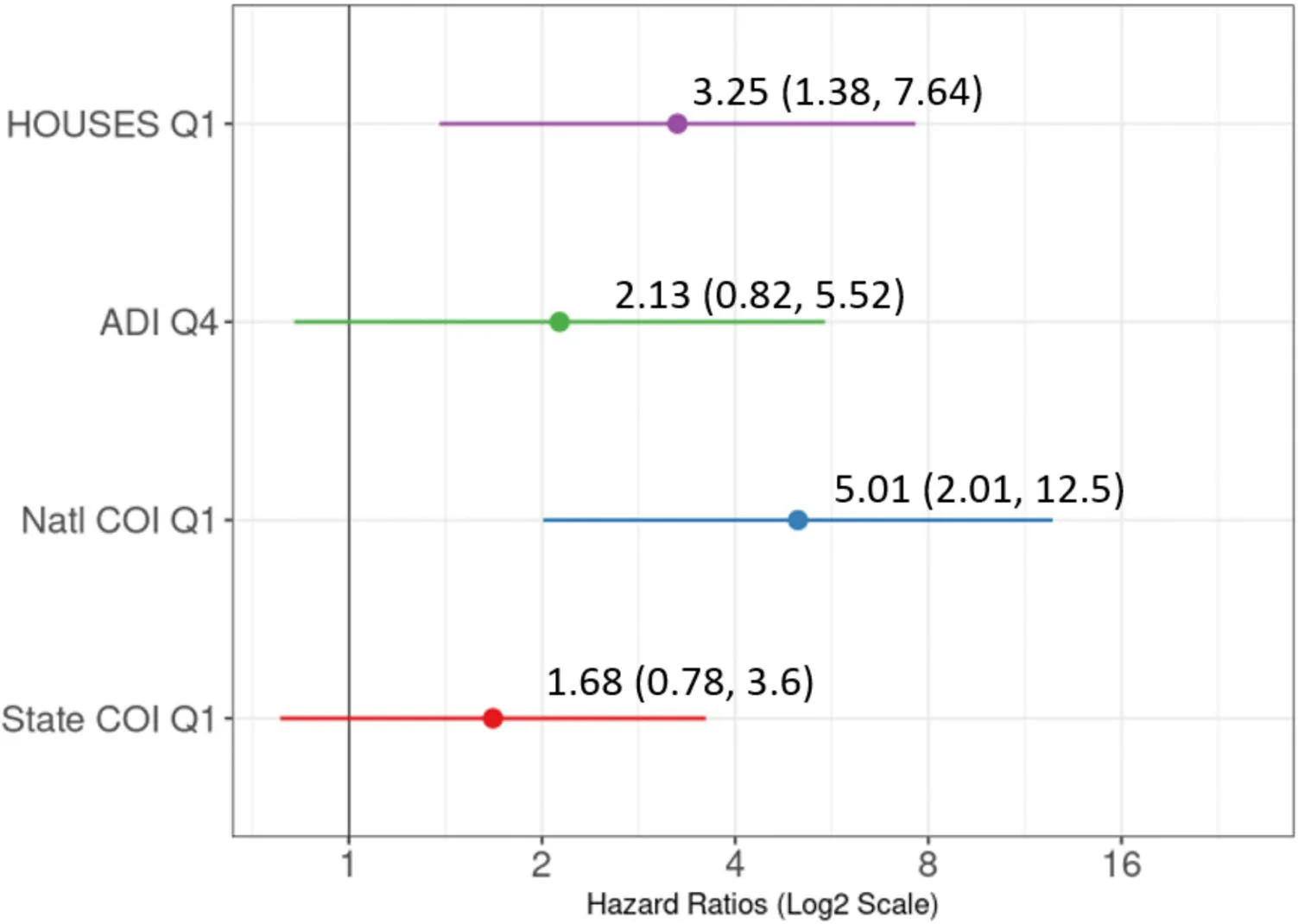
Forest plot

We found no significant effect of HOUSES on graft survival on univariate analysis (HR: 1.63; 95% CI: 0.79, 3.35; *p* = 0.18). However, after adjusting for age at transplant, sex, donor type, pretransplant dialysis, insurance, cause of kidney failure, and BMI z-scores, we observed that Q1 recipients (low SES) were at significantly increased graft loss risk compared to Q2–4 recipients (high SES) (Adjusted Hazard Ratio [aHR]: 3.25; 95% CI: 1.38, 7.64; *p* = 0.007) (Fig. 2).

The causes of graft loss are shown in Table 3.

### COI_NAT_ and graft survival

We observed significantly higher rates of delayed graft function in low COI_NAT_ recipients compared to high COI_NAT_ recipients on adjusted analysis (Low COI_NAT_ vs. high COI_NAT_: 13.0% vs. 1.8%; *p* = 0.008). Graft survival by COI_NAT_ is shown in Fig. 1.

We observed a significantly lower graft survival in recipients with low COI_NAT_ compared to those with high COI_NAT_ (HR: 4.58; 95% CI: 2.06, 10.17; *p* < 0.001). The association remained statistically significant after adjusting for covariates (aHR: 5.01; 95% CI: 2.01, 12.5; *p* = 0.001).

Forty percent of recipients in the low COI_NAT_ group lost their graft to acute rejection compared to only 8.3% of recipients in the high COI_NAT_ group (Table 3).

### COI_ST_ and graft survival

We found no difference in delayed graft function rates between low COI_ST_ and high COI_ST_ groups (4.5% vs. 2.8%; *p* = 0.59). Figure 1 compares graft survival between low COI_ST_ and high COI_ST_ groups (Fig. 1).

We observed no statistically significant association between COI_ST_ and graft survival on univariate (HR: 1.38; 95% CI: 0.7, 2.7; *p* = 0.354) or multivariable analysis (aHR: 1.68; 95% CI: 0.78, 3.6; *p* = 0.18).

In terms of the causes of graft loss, 27.8% of recipients with low COI_ST_ lost their graft to acute rejection compared to 6.3% of recipients with high COI_ST_ (Table 3).

### ADI and graft survival

We observed no difference in delayed graft function rates between high-deprivation ADI and low-deprivation ADI groups (6.2% vs. 3.3%; *p* = 0.55). Figure 1 compares graft survival between high-deprivation ADI and low-deprivation ADI groups.

We observed no statistically significant association between ADI and graft survival on univariate (HR: 1.48 (0.61, 3.58); *p* = 0.3.87) or multivariable analysis (aHR: 2.13; 95% CI: 0.82, 5.52; *p* = 0.12).

Fifty percent of recipients in the high-deprivation ADI group lost their graft to acute rejection compared to 10.7% of those in the low-deprivation ADI group (Table 3).

## Discussion

Our study demonstrates significant SES-related disparities in pediatric kidney transplant recipients. Furthermore, it highlights that the choice of SES metric may influence the interpretation of the impact of SES on health outcomes in this population. While COI_NAT_ was a significant predictor of graft loss in our cohort, we found no association between graft loss and other widely used indices, specifically the COI_ST_ and ADI. Our findings indicate that certain area-level SES measures, if used solely as proxies for individual-level SES, may fail to detect the true impact of SES on graft outcomes. Our findings urge a systematic approach toward identifying vulnerable individuals and implementing targeted interventions to mitigate disparities.

Racial disparities in graft outcomes among pediatric kidney transplant recipients are well documented; Black pediatric kidney transplant recipients consistently demonstrate lower short- and long-term graft survival compared to White recipients [7, 8]. As race in the US is closely linked to SES [9], it is important to elucidate the role of SES in the relationship between race and transplant outcomes. A retrospective study of 6216 children with kidney failure, transplanted between 2000 and 2011, failed to detect a significant interaction between race and SES, with Black recipients experiencing lower graft survival in both low and high-income neighborhoods and with both public and private insurance [7]. However, the study relied on neighborhood measures of SES, which may have misclassified individual-level SES. Our study is the first to use the HOUSES Index, an individual-level SES measure, to determine its association with graft survival in children. We observed a threefold higher risk of graft loss in children with a low HOUSES quartile, after adjusting for covariates. Notably, HOUSES was not associated with graft loss on univariate analysis, indicating that covariates were masking the true association between HOUSES and graft loss. Recognizing that obesity is a risk factor for graft loss in pediatric kidney transplant recipients [28] and disproportionately affects children with lower SES [29], we adjusted our model for BMI z-scores in addition to other clinically relevant covariates, revealing an independent association between HOUSES and graft loss.

While the COI_NAT_ predicted graft loss in our cohort, COI_ST_ did not. The superior performance of COI_NAT_ compared to COI_ST_ may be explained by the unique socioeconomic landscape of Minnesota. As an affluent state with a robust healthcare infrastructure [30], the state-level neighborhood indices for Minnesota may be skewed toward a higher baseline compared to the national average. Hence, a low COI_ST_ may not capture the threshold of deprivation that impedes care access and delivery. In contrast, COI_NAT_ benchmarks neighborhoods against the national average, which includes states with substantially fewer resources. Hence, COI_NAT_ identifies those facing extreme deprivation in Minnesota. This is evidenced by the fact that of the 66 recipients classified in the lowest COI_ST_ quartile, only 23 were captured by the lowest COI_NAT_ quartile. The relative privilege of children in Minnesota is also indicated by the fact that nearly 60% of our cohort had private insurance, as 61.3% of children in Minnesota are on an employer-sponsored insurance plan [31]. Our findings suggest that in high-resource regions, state-based indices may lack the granularity necessary to identify the most high-risk individuals, and nationally standardized indices may serve as better measures of the degree of deprivation. Furthermore, since COI is based on regional resources rather than individual assets, these measures may not accurately identify recipients for individual-level targeted interventions.

We observed no association between neighborhood ADI and graft survival in our study. Consistent with our findings, a retrospective, single-center pediatric study of 78 kidney transplant recipients by Chen et al. found no effect of ADI on graft survival [32]. Furthermore, a retrospective study of 2972 children residing in 108 US counties found no association between a county-level SES measure and 1-year graft survival [14]. The inability of certain area-level SES measures to identify the association between SES and graft outcomes is likely due to the misclassification of individual-level SES inherent in area-level measures [15, 16]. Conversely, certain area-level measures may effectively capture factors that hamper healthcare access, such as the COI_NAT_ in our study. Consistent with our COI_NAT_ findings, a retrospective study of 9,178 children listed for transplant before 18 years of age found a 1.5-fold increased risk of graft loss in children with high neighborhood deprivation, determined by the Material Community Deprivation Index score [13]. However, the strength of association observed in our cohort was substantially higher (aHR: 5.01) likely because COI_NAT_ captured more extreme levels of deprivation in the Minnesota context.

Adult studies examining the role of SES in kidney transplant outcomes yield mixed results. Some studies find private insurance, higher level of education, and skilled occupation to be associated with improved graft survival [33–35], while other studies find no association between SES and KT outcomes, likely due to using area-level SES measures [36–38]. Aligned with our findings, a previous single-center adult study using the HOUSES Index found significantly lower graft survival in recipients with lower SES [13]. Furthermore, in an Olmsted County, MN, population-based retrospective cohort study of adult recipients, Steven et al. demonstrated lower graft survival in recipients with lower SES using the HOUSES Index. Notably, and consistent with our findings, Steven et al. found no significant effect of area-level SES measures or educational attainment on graft survival [19].

SES likely exerts its effects on graft survival through several interlinked mechanisms. At the individual level, outcomes may be driven by access barriers, such as missed clinic appointments due to inadequate transportation and medication non-adherence resulting from limited health literacy or reduced supervision when caregivers work extended hours [11]. Additionally, competing priorities like food and housing insecurity may take precedence over medical care. At the neighborhood level, limited infrastructure may lead to poor access to pharmacies, nutritious food, and reliable public transportation. High levels of crime and a lack of community support may jeopardize physical safety and mental health. Furthermore, chronic SES-mediated stressors may impair executive functioning, which is critical for managing complex post-transplant regimens, particularly in adolescents [39]. Collectively, these multifaceted challenges disrupt treatment, culminating in premature graft decline.

Incorporating SES into risk prediction models may enable more personalized posttransplant monitoring and tailored care strategies, including closer follow-up, adherence support, and health literacy-appropriate education. For instance, smart devices administered to recipients in the low SES group could increase engagement by providing an interactive platform for: managing medication lists and appointments, setting fluid goals, requesting medication refills, receiving alerts, and tracking task completion [40]. HOUSES and COI_NAT_ could be used to identify recipients most in need of transportation support, mobile phlebotomy, and mail-order pharmacies. Community-based initiatives, such as cultural liaisons and home nurse visits, could be prioritized for recipients with low SES to promote culturally competent care and to build trust and bridge communication gaps. Similarly, resources could be prioritized to address lost wages and childcare needs that hinder medical appointments. Furthermore, HOUSES and COI_NAT_ could be used to measure SES in prospective studies designed to evaluate the effectiveness of above-listed interventions in mitigating disparities.

Our study had several limitations. Given the retrospective nature, we could not account for unknown confounders. The results of our single-center study may not be generalizable to centers with different patient demographics. Similarly, the performance of COI_NAT_ in Minnesota may not apply to states with fewer resources. Furthermore, center-specific differences in policies and resources may exacerbate or mitigate socioeconomic challenges. Nevertheless, this is an important study as it illustrates the role of SES in pediatric transplant outcomes despite regional affluence and robust healthcare infrastructure. A multicenter national study is necessary to validate these associations across diverse geographic regions, overcoming the limitations of our single-center study design.

In summary, this study identifies SES as a significant risk factor for graft loss in pediatric kidney transplant recipients, demonstrating that regional affluence does not insulate vulnerable recipients from adverse outcomes. The HOUSES Index was associated with a threefold increased risk of graft loss, and COI_NAT_ with a fivefold increased risk. While the magnitude of this association is concerning, it identifies a clear opportunity for intervention, as unlike biological risk factors, SES-mediated risk can be mitigated through targeted interventions. “Low-hanging fruit” such as mail order pharmacies, mobile phlebotomies, and virtual clinic visits could substantially alleviate the burden of care for these families. Future studies should evaluate the effectiveness of such interventions in improving outcomes. Given the finite nature of resources, the HOUSES Index and COI_NAT_ could be useful tools to identify recipients most in need of interventions, paving the path for eliminating disparities in pediatric kidney transplantation.

## Supplementary Material

MOESM3

MOESM2

ESM

**Supplementary Information** The online version contains supplementary material available at https://doi.org/10.1007/s00467-026-07322-6.

## Figures and Tables

**Table 1:** Patient characteristics by HOUSES

	Q1 (*N* = 38)	Q2–4 (*N* = 99)	*p* value

Age at transplant			0.882^a^
Mean (SD)	11.782 (5.414)	11.639 (4.889)	
Median (Q1, Q3)	13.150 (7.473, 16.540)	12.630 (7.520, 15.545)	
Range	2.060–19.610	2.130–18.460	
Sex			0.343^b^
F	15 (39.5%)	48 (48.5%)	
M	23 (60.5%)	51 (51.5%)	
Race			
White	25 (65.8%)	78 (78.8%)	
Black	4 (10.5%)	8 (8.1%)	
Asian	7 (18.4%)	10 (10.1%)	
American Indian or Alaska Native	2 (5.3%)	3 (3.0%)	
Other	0 (0.0%)	0 (0.0%)	
Ethnicity			0.421^b^
(Missing)	2 (5.3%)	10 (10.1%)	
Hispanic/Latino	3 (7.9%)	13 (13.1%)	
Non-Hispanic/NonLatino	33 (86.8%)	76 (76.8%)	
Race/ethnicity			0.453^b^
(Missing)	0 (0.0%)	4 (4.0%)	
Non-Hispanic White	23 (60.5%)	57 (57.6%)	
Other	15 (39.5%)	38 (38.4%)	
ADI binary group			
1–3	33 (86.8%)	88 (88.9%)	
4	5 (13.2%)	11 (11.1%)	
(Missing)	0 (0.0%)	0 (0.0%)	
COI national binary group			
1	11 (28.9%)	12 (12.1%)	
2–4	27 (71.1%)	87 (87.9%)	
(Missing)	0 (0.0%)	0 (0.0%)	
COI state binary group			
1	23 (60.5%)	43 (43.4%)	
2–4	15 (39.5%)	56 (56.6%)	
(Missing)	0 (0.0%)	0 (0.0%)	
Binary insurance			0.050^b^
Private	18 (47.4%)	65 (65.7%)	
Public/other	20 (52.6%)	34 (34.3%)	
Pretransplant dialysis			0.011^b^
N	5 (13.2%)	35 (35.4%)	
Y	33 (86.8%)	64 (64.6%)	
Time on dialysis			0.011^b^
Not on dialysis	5 (13.2%)	35 (35.4%)	
< 1 year	18 (47.4%)	39 (39.4%)	
1–3 years	13 (34.2%)	16 (16.2%)	
> 3 years	2 (5.3%)	2 (2.0%)	
Unknown time on dialysis	0 (0.0%)	7 (7.1%)	
Donor type			0.145^b^
Deceased	21 (55.3%)	41 (41.4%)	
Living	17 (44.7%)	58 (58.6%)	
HLA mismatch			0.370^a^
N-Miss	0	4	
Mean (SD)	3.605 (1.717)	3.863 (1.396)	
Median (Q1, Q3)	4.000 (2.000, 5.000)	4.000 (3.000, 5.000)	
Range	0.000–6.000	1.000–6.000	
CDC BMI z-score			0.227^a^
Mean (SD)	−0.192 (1.423)	0.122 (1.335)	
Median (Q1, Q3)	−0.268 (−0.955, 0.919)	0.242 (−0.706, 1.127)	
Range	−4.053–2.114	−3.619–2.445	
Cause of kidney failure			0.597^b^
Congenital anomalies	15 (39.5%)	46 (46.5%)	
FSGS	5 (13.2%)	20 (20.2%)	
Glomerulonephritis	2 (5.3%)	5 (5.1%)	
Other	12 (31.6%)	22 (22.2%)	
Unknown	4 (10.5%)	6 (6.1%)	

a Linear model ANOVA

b Pearson’s chi-squared test

**Table 2:** Patient characteristics by COI_NAT_

	1 (*N* = 23)	2–4 (*N* = 114)	*p* value

Age at transplant			0.564^a^
Mean (SD)	12.232 (5.578)	11.567 (4.919)	
Median (Q1, Q3)	13.410 (7.665, 16.970)	12.495 (7.400, 15.747)	
Range	2.190–19.610	2.060–19.330	
Sex			0.266^b^
F	13 (56.5%)	50 (43.9%)	
M	10 (43.5%)	64 (56.1%)	
Race			
White	10 (43.5%)	93 (81.6%)	
Black	3 (13.0%)	9 (7.9%)	
Asian	9 (39.1%)	8 (7.0%)	
American Indian or Alaska Native	1 (4.3%)	4 (3.5%)	
Other	0 (0.0%)	0 (0.0%)	
HOUSES binary group			
1	11 (47.8%)	27 (23.7%)	
2–4	12 (52.2%)	87 (76.3%)	
(Missing)	0 (0.0%)	0 (0.0%)	
ADI binary group			
1–3	18 (78.3%)	103 (90.4%)	
4	5 (21.7%)	11 (9.6%)	
(Missing)	0 (0.0%)	0 (0.0%)	
COI state binary group			
1	23 (100.0%)	43 (37.7%)	
2–4	0 (0.0%)	71 (62.3%)	
(Missing)	0 (0.0%)	0 (0.0%)	
Binary insurance			0.006^b^
Private	8 (34.8%)	75 (65.8%)	
Public/other	15 (65.2%)	39 (34.2%)	
Pretransplant dialysis			0.062^b^
N	3 (13.0%)	37 (32.5%)	
Y	20 (87.0%)	77 (67.5%)	
Time on dialysis			0.001^b^
Not on dialysis	3 (13.0%)	37 (32.5%)	
< 1 year	5 (21.7%)	52 (45.6%)	
1–3 years	11 (47.8%)	18 (15.8%)	
> 3 years	1 (4.3%)	3 (2.6%)	
Unknown time on dialysis	3 (13.0%)	4 (3.5%)	
Donor type			0.035^b^
Deceased	15 (65.2%)	47 (41.2%)	
Living	8 (34.8%)	67 (58.8%)	
HLA mismatch			0.778^a^
N-Miss	0	4	
Mean (SD)	3.870 (1.486)	3.773 (1.500)	
Median (Q1, Q3)	4.000 (3.000, 5.000)	4.000 (3.000, 5.000)	
Range	0.000–6.000	0.000–6.000	
CDC BMI z-score			0.656^a^
Mean (SD)	0.151 (1.087)	0.012 (1.414)	
Median (Q1, Q3)	−0.316 (−0.759, 0.970)	0.172 (−0.792, 1.119)	
Range	−1.279–2.114	−4.053–2.445	
Cause of kidney failure			0.039^b^
Congenital anomalies	4 (17.4%)	57 (50.0%)	
FSGS	6 (26.1%)	19 (16.7%)	
Glomerulonephritis	3 (13.0%)	4 (3.5%)	
Other	8 (34.8%)	26 (22.8%)	
Unknown	2 (8.7%)	8 (7.0%)	

a Linear model ANOVA

b Pearson’s chi-squared test

**Table 3:** Causes of graft loss by SES

HOUSES			
	HOUSES Q1 *n* = 11	HOUSES Q2-4 *n* = 23	0.40
Acute rejection	3 (27.3%)	3 (13.0%)	
Chronic rejection	2 (18.2%)	5 (21.7%)	
Graft thrombosis	1 (9.1%)	0 (0.0%)	
Infection	1 (9.1%)	1 (4.3%)	
Other	2 (18.2%)	10 (43.5%)	
Primary graft failure	0 (0.0%)	2 (8.7%)	
Recurrent disease	2 (18.2%)	2 (8.7%)	
COI_NAT_			
	Low opportunity COI_NAT_ *n* = 10	High opportunity COI_NAT_ *n* = 24	0.35
Acute rejection	4 (40.0%)	2 (8.3%)	
Chronic rejection	2 (20.0%)	5 (20.8%)	
Graft thrombosis	0 (0.0%)	1 (4.2%)	
Infection	0 (0.0%)	2 (8.3%)	
Other	4 (40.0%)	8 (33.3%)	
Primary graft failure	0 (0.0%)	2 (8.3%)	
Recurrent disease	0 (0.0%)	4 (16.7%)	
COI_ST_			
	Low opportunity COI_ST_ *n* = 18	High opportunity COI_ST_ *n* = 16	0.10
Acute rejection	5 (27.8%)	1 (6.3%)	
Chronic rejection	3 (16.7%)	4 (25.0%)	
Graft thrombosis	1 (5.5%)	0 (0.0%)	
Infection	2 (11.1%)	0 (0.0%)	
Other	6 (33.3%)	6 (37.5%)	
Primary graft failure	1 (5.5%)	1 (6.3%)	
Recurrent disease	0 (0.0%)	4 (25.0%)	
ADI			
	Low deprivation *n* = 28	High deprivation *n* = 6	0.28
Acute rejection	3 (10.7%)	3 (50.0%)	
Chronic rejection	7 (25.0%)	0 (0.0%)	
Graft thrombosis	1 (3.6%)	0 (0.0%)	
Infection	2 (7.1%)	0 (0.0%)	
Other	9 (32.1%)	3 (50.0%)	
Primary graft failure	2 (7.1%)	0 (0.0%)	
Recurrent disease	4 (14.3%)	0 (0.0%)	

## Data Availability

Data is available from the authors on request.
