# Supplementary material for: Beyond biological risk: socioeconomic vulnerability and adverse pediatric kidney transplant outcomes
Source: Pediatr Nephrol. Author manuscript; Available in PMC 2026 Aug 21. (PMC13481395; doi:10.1007/s00467-026-07322-6)
Supplement: Supplementary file 1 [file NIHMS2199671-supplement-MOESM2.docx]

**Supplementary tables**

| **Table 1. Publications showing health disparities by HOUSES (adults) (39 outcomes)** | | |
| --- | --- | --- |
| **Study** | **Outcomes** | **Adjusted effect size for Q1 (lowest SES by HOUSES index) (95% CI)^a^** |
| **1. Chronic conditions** | | |
| Ahn, 2023^1^ | **Alcohol-associated hepatitis** | Distribution of HOUSES: 39.9% (Q1), 12.5%% (Q2), 12.3% (Q3), and 17.1% (Q4) |
| Tadese, 2022^2^ | **Severe obesity** | OR = 1.33 (0.99–1.80) |
| Greenwood, 2022^3^ | **Diabetic control** | OR = 0.83 (0.70–0.98) |
| Rusk, 2022^4^ | **Indigenous Smoking Behaviors** | Distribution of HOUSES in indigenous cohort (vs. match cohort): 39.9% (vs. 26.7%) for Q1, 12.5%% (vs. 24.3%) for Q2, 10.4% (vs. 20.6%) for Q3, and 8.0% (vs. 24.8%) for Q4 |
| Rusk, 2022^5^ | **Smoking Behaviors Among Indigenous Pregnant People** | Distribution of HOUSES in indigenous cohort (vs. match cohort): 40.4% (vs. 35.2%) for Q1, 33.3% (vs. 28.1%) for Q2, 12.3% (vs. 23.0%) for Q3, and 14.0% (vs. 13.8%) for Q4 |
| Angstman, 2021^6^ | **Persistent depressive symptoms** | OR = 1.31 (1.06–1.61) |
|  | **Remission of depressive symptoms** | OR = 0.78 (0.65–0.95) |
| Thacher, 2020^7^ | **25-Hydroxyvitamin D Level** | Estimate = 0.28 (0.21–0.35) |
| Stevens, 2020^8^ | **Kidney transplantation failure** | HR = 2.0 (1.04–3.84) |
| Ryan, 2020^9^ | **Post-Glioma mortality** | HR = 1.61 (1.05–2.5) |
| Patten, 2020^10^ | **Mood disorder and obesity** | Distribution of HOUSES in rural (vs. urban):  11% (vs. 28%) for Q1, 21% (vs. 27%) for Q2, 28% (vs. 25%) for Q3, 40% (20%) for Q4 |
| Takahashi, 2016^11^ | **All-cause hospitalization** | HR = 1.53 (1.18–1.98) |
|  | **Multiple chronic conditions prevalence** | OR = 2.4 (2.0–3.1) |
| Wi, 2016^12^ | **Coronary heart disease prevalence** | OR = 1.35 (1.25–1.44) |
|  | **Asthma prevalence** | OR = 1.28 (1.20–1.36) |
|  | **Diabetes prevalence** | OR = 1.78 (1.66–1.92) |
|  | **Hypertension prevalence** | OR = 1.56 (1.47–1.63) |
|  | **Mood disorder prevalence** | OR = 1.63 (1.58–1.72) |
| Ghawi, 2015^13^ | **Rheumatoid Arthritis incidence** | OR = 1.06 (1.02–1.09) |
|  | **Post-Rheumatoid Arthritis mortality** | HR = 1.58 (1.05–2.36) |
| Bang, 2014^14^ | **Post–Myocardial Infarction mortality** | HR = 1.86 (1.07–3.24) |
| **2. Acute conditions** | | |
| Wi, 2023^15^ | **Subject recruitment** | Distribution of HOUSES in Standard mailing (TESRS): 16.1% (vs. 12.9% for Q1, 9.7 (vs. 12.9%) for Q2, 45.2% (vs. 45.2%) for Q3, and 29.0% (vs. 29.0%) for Q4 |
| Zurek, 2022^16^ | **Hospital readmission** | OR* = 0.90 (0.83–0.98) for Q3 |
| Vachon, 2022^17^ | **COVID-19 asymptomatic rates** | HR* = 1.22 (0.86–1.7) for Q4 |
| Juhn 2021^18^ | **COVID-19 incidence in urban** | Identified geographical hotspots taking into account HOUSES |
| Wheeler 2021^19^ | **COVID-19 incidence in rural** | Identified geographical hotspots taking into account HOUSES |
| Aul, 2020^20^ | **Osteoporotic fracture incidence** | HR = 1.05 (1.04–1.08) |
| Barwise, 2020^21^ | **Mortality rates (>50 years of age)** | HR = 1.38 (1.07–1.78) |
| Ryu, 2017^22^ | **Accidental falls incidence** | HR = 1.72 (1.31–2.27) |
| **3. Behavioral risk factors and others** | | |
| Felzer, 2023^23^ | **Influenza vaccination in solid organ transplant patients** | RR* = 1.14 (1.008–1.30) for Q3 |
| Juhn, 2021^24^ | **Adherence to Public Health Measures Mitigates the Risk of COVID-19 Infection** | Used as a covariate for basic characteristics  12.3% (Q1), 25.5% (Q2), 28.3% (Q3), and 33.9% (Q4) |
| MacLaughlin, 2020^25^ | **HPV Vaccination (initiation and completion)** | RR = 1.15 [1.03–1.28] for Q4 (initiation)  RR = 1.32 [1.21–1.44] for Q4 (completion) |
| Barwise, 2019^26^ | **Advance Care**  **Planning and Nursing Home Utilization** | OR* = 0.77 (0.63–0.93) for advance directives  OR* = 0.60 (0.50–0.72) for discharge to home |
| Ryu, 2018^27^ | **Inconsistency of self-reported disease prevalence in survey results** | OR = 1.46 (1.17–1.84) |
| Barwise, 2018^28^ | **Rates of social work consultation in end-of-life care** | OR = 1.46 (1.18–1.79) |
|  | **Advance directives** | OR =1.29 (1.07–1.58) |
| Wi, 2016^29^ | **Smoking status** | OR = 2.56 (1.14–5.55) |
| ^a^ Highest HOUSES group as a reference, except Zurek et al 2022* and Felzer et al 2022* which used lowest HOUSES group, Vachon et al 2022* which used 2^nd^ quartile of HOUSES group, and Barwise et al 2019* which used 2^nd^–4^th^ quartile of HOUSES group as a reference | | |

| **Table 2. Publications showing health disparities by HOUSES (children) (23 outcomes)** | | | | |
| --- | --- | --- | --- | --- |
| **Study** | **Outcomes** | | **Adjusted effect size for Q1 (lowest SES by HOUSES index) (95% CI)^a^** | |
| **1. Chronic conditions** | | | | |
| Skolnick 2023^30^ | **Severe Obesity** | | OR = 2.78 (1.91–4.06) | |
| Rodriguez, 2023^31^ | **Acne prevalence** | | Distribution of HOUSES in acne cases (vs. matched controls): 42.2% (vs. 32.7%) for Q1, 22.8% (vs. 25.5%) for Q2, 17.4% (vs. 20.1%) for Q3, and 17.5% (21.7%) for Q4 | |
| Bjur 2019^32^ | **Asthma prevalence** | | OR = 1.14 (1.03–1.29) | |
|  | **Epilepsy prevalence** | | OR = 1.88 (1.21–2.85) | |
|  | **Mood disorders prevalence** | | OR =1.38 (1.19–1.61) | |
| Bjur 2019^33^ | **Multiple complex chronic conditions prevalence** | | 5-year prevalence in 2004:  Q1 (1,443), Q2 (1,435), Q3 (1,134), Q4 (1,124) | |
| Ryu 2016^34^ | **Overweight prevalence** | | OR = 2.08 (1.06–4) (OC) **^b^**  OR = 2.22 (1.12–4.34) (JC) **^b^** | |
|  | **Low birth weight prevalence** | | OR = 1.28 (0.49–3.33) (OC) **^b^**  OR = 1.85 (0.82–2.94) (JC) **^b^** | |
|  | **Household smoking status** | | OR = 4.16 (2.04–9.09) (OC) **^b^**  OR = 3.84 (2.27–6.25) (JC) **^b^** | |
| Lynch 2015^35^ | **Overweight/Obesity**  **(HOUSES as covariate)** | | Mean (SD) (p<0.0001)  Healthy weight: 1.7 (3.9)  Overweight: 1.0 (3.8)  Obesity: 0.1 (3.4) | |
| Harris 2014^36^ | **Poorly controlled asthma status** | | OR = 4.76 (1.12–20) | |
| Butterfield 2011^37^ | **Adverse self-rated health** | | OR = 4.76 (1.96–12.5) (OC) **^b^**  OR = 1.01 (0.24–4.0) (JC) **^b^** | |
| Juhn 2011^38^ | **Overweight prevalence** | | OR=2.56 (p=0.008) (OC) **^b^**  OR=2.04 (p=0.07) (JC) **^b^** | |
|  | **Low birth weight prevalence** | | OR=1.58 (p=0.330) (OC) **^b^**  OR=2.38 (p=0.018) (JC) **^b^** | |
|  | **Household smoking status prevalence** | | OR=2.56 (p=0.007) (OC) **^b^**  OR=4.16 (p<.001) (JC) **^b^** | |
| **2. Acute conditions** | | | | |
| Patel 2020^22^ | | **Adverse childhood events risk**  **(HOUSES as covariate)** | | Proportion of outcomes by HOUSES quartile  Q1 (4.6%), Q2 (3.5%), Q3 (3.0%), Q4 (2.1%) |
| Bjur 2019^32^ | **Bronchiolitis prevalence** | | OR = 1.44 (1.28–1.63) | |
|  | **Pneumonia prevalence** | | OR = 1.13 (1.01–1.28) | |
|  | **Urinary Tract Infection prevalence** | | OR = 1.51 (1.28–1.78) | |
|  | **Adverse childhood experiences prevalence** | | OR = 1.85 (1.51–2.27) | |
| Johnson 2013^39^ | **Invasive pneumococcal disease risk** | | OR = 4.54 (1.12–20) | |
| **3. Behavioral risk factors** | | | | |
| Maclaughlin 2020^25^ | **HPV vaccine initiation** | | Rates: 0.86 (0.78–0.97) | |
|  | **HPV vaccine completion** | | Rates: 0.75 (0.69–0.82) | |
| Hammer 2016^40^ | **Pertussis vaccine up-to-date status** | | OR = 0.27 (0.10–0.74) | |
| **4. AI bias** | | | | |
| Juhn 2022^41^ | **Performance of predicting risk of asthma exacerbation** | | Balance error rate [(FPR+FNR)/2]  Q1 = 0.53, Q2–4: 0.39 (Ratio: 1.35) | |
| **^a^** Highest HOUSES group as a reference; **^b^** OC: Olmsted county, Minnesota JC: Jackson county, Missouri | | | | |

1.	Ahn JC, Wi CI, Buryska S, et al. Disproportionate increases in alcohol-associated hepatitis incidence in women and individuals of low socioeconomic status: A population-based study using the Rochester epidemiology project database. Hepatology Communications 2023;7.

2.	Tadese K, Ernst V, Weaver AL, et al. Association of Perinatal Factors With Severe Obesity and Dyslipidemia in Adulthood. J Prim Care Community Health 2022;13:21501327211058982.

3.	Greenwood J, Zurek KI, Grimm JM, et al. Association of a housing based individual socioeconomic status measure with diabetic control in primary care practices. Prim Care Diabetes 2022;16:78-83.

4.	Rusk AM, Giblon RE, Chamberlain AM, et al. Indigenous Smoking Behaviors in Olmsted County, Minnesota: A Longitudinal Population-Based Study. Mayo Clinic Proceedings 2022;97:1836-1848.

5.	Rusk AM, Giblon RE, Chamberlain AM, et al. Smoking Behaviors Among Indigenous Pregnant People Compared to a Matched Regional Cohort. Nicotine & Tobacco Research 2023;25:889-897.

6.	Angstman KB, Wi CI, Williams MD, et al. Impact of socioeconomic status on depression clinical outcomes at six months in a Midwestern, United States community. Journal of Affective Disorders 2021;292:751-756.

7.	Thacher TD, Dudenkov DV, Mara KC, et al. The relationship of 25-hydroxyvitamin D concentrations and individual-level socioeconomic status. J Steroid Biochem Mol Biol 2020;197:105545.

8.	Stevens MA, Beebe TJ, Wi C-I, et al. HOUSES index as an innovative socioeconomic measure predicts graft failure among kidney transplant recipients. Transplantation 2020;Online First.

9.	Ryan CS, Juhn YJ, Kaur H, et al. Long-term incidence of glioma in Olmsted County, Minnesota, and disparities in postglioma survival rate: a population-based study. Neurooncol Pract 2020;7:288-298.

10.	Patten CA, Juhn YJ, Ryu E, et al. Rural-urban health disparities for mood disorders and obesity in a midwestern community. Journal of Clinical and Translational Science 2020;4:408-415.

11.	Takahashi PY, Ryu E, Hathcock MA, et al. A novel housing-based socioeconomic measure predicts hospitalisation and multiple chronic conditions in a community population. J Epidemiol Community Health 2016;70:286-91.

12.	Wi CI, St Sauver JL, Jacobson DJ, et al. Ethnicity, Socioeconomic Status, and Health Disparities in a Mixed Rural-Urban US Community-Olmsted County, Minnesota. Mayo Clin Proc 2016;91:612-22.

13.	Ghawi H, Crowson CS, Rand-Weaver J, et al. A novel measure of socioeconomic status using individual housing data to assess the association of SES with rheumatoid arthritis and its mortality: a population-based case-control study. BMJ Open 2015;5:e006469.

14.	Bang DW, Manemann SM, Gerber Y, et al. A novel socioeconomic measure using individual housing data in cardiovascular outcome research. Int J Environ Res Public Health 2014;11:11597-615.

15.	Wi CI, King KS, Ryu E, et al. Application of Innovative Subject Recruitment System for Batch Enrollment: A Pilot Study. Journal of Primary Care and Community Health 2023;14.

16.	Zurek KI, Boswell CL, N EM, et al. Association of Early and Late Hospital Readmissions with a Novel Housing-Based Socioeconomic Measure. Health Serv Res Manag Epidemiol 2022;9:23333928221104644.

17.	Vachon CM, Norman AD, Prasad K, et al. Rates of Asymptomatic COVID-19 Infection and Associated Factors in Olmsted County, Minnesota, in the Prevaccination Era. Mayo Clin Proc Innov Qual Outcomes 2022;6:605-617.

18.	Juhn YJ, Wheeler P, Wi CI, et al. Role of Geographic Risk Factors in COVID-19 Epidemiology: Longitudinal Geospatial Analysis. Mayo Clin Proc Innov Qual Outcomes 2021;5:916-927.

19.	Wheeler PH, Patten CA, Wi CI, et al. Role of geographic risk factors and social determinants of health in COVID-19 epidemiology: Longitudinal geospatial analysis in a midwest rural region. Journal of Clinical and Translational Science 2021;6.

20.	Aul AJ, Dudenkov DV, Mara KC, et al. The relationship of 25-hydroxyvitamin D values and risk of fracture: a population-based retrospective cohort study. Osteoporos Int 2020;31:1787-1799.

21.	Barwise A, Wi CI, Frank R, et al. An Innovative Individual-Level Socioeconomic Measure Predicts Critical Care Outcomes in Older Adults: A Population-Based Study. J Intensive Care Med 2020:885066620931020.

22.	Ryu E, Juhn YJ, Wheeler PH, et al. Individual housing-based socioeconomic status predicts risk of accidental falls among adults. Ann Epidemiol 2017;27:415-420.e2.

23.	Felzer JR, Rutten LFJ, Wi CI, et al. Disparities in vaccination rates in solid organ transplant patients. Transplant Infectious Disease 2023;25.

24.	Juhn YJ, Wi CI, Ryu E, et al. Adherence to Public Health Measures Mitigates the Risk of COVID-19 Infection in Older Adults: A Community-Based Study. Mayo Clin Proc 2021;96:912-920.

25.	MacLaughlin KL, Jacobson RM, Sauver JLS, et al. An innovative housing-related measure for individual socioeconomic status and human papillomavirus vaccination coverage: A population-based cross-sectional study. Vaccine 2020;38:6112-6119.

26.	Barwise A, Juhn YJ, Wi CI, et al. An Individual Housing-Based Socioeconomic Status Measure Predicts Advance Care Planning and Nursing Home Utilization. American Journal of Hospice & Palliative Medicine 2019;36:362-369.

27.	Ryu E, Olson JE, Juhn YJ, et al. Association between an individual housing-based socioeconomic index and inconsistent self-reporting of health conditions: a prospective cohort study in the Mayo Clinic Biobank. BMJ Open 2018;8:e020054.

28.	Barwise A, Juhn YJ, Wi CI, et al. An Individual Housing-Based Socioeconomic Status Measure Predicts Advance Care Planning and Nursing Home Utilization. Am J Hosp Palliat Care 2018.

29.	Wi CI, Gauger J, Bachman M, et al. Role of individual-housing-based socioeconomic status measure in relation to smoking status among late adolescents with asthma. Ann Epidemiol 2016;26:455-60.

30.	Skolnick V, Rajjo T, Thacher T, et al. Association of Weight Trajectory with Severe Obesity: A Case-Control Study. Childhood Obesity 2023.

31.	Baisi KRE, Weaver AL, Wi CI, et al. Socioeconomic status, race, and preadolescent acne: A population-based retrospective cohort analysis in a mixed rural-urban community of the United States (Olmsted County, Minnesota). Pediatric Dermatology 2023;40:460-465.

32.	Bjur KA, Wi CI, Ryu E, et al. Socioeconomic Status, Race/Ethnicity, and Health Disparities in Children and Adolescents in a Mixed Rural-Urban Community-Olmsted County, Minnesota. Mayo Clin Proc 2019;94:44-53.

33.	Bjur KA, Wi CI, Ryu E, et al. Epidemiology of Children With Multiple Complex Chronic Conditions in a Mixed Urban-Rural US Community. Hosp Pediatr 2019;9:281-290.

34.	Ryu E, Wi CI, Crow SS, et al. Assessing health disparities in children using a modified housing-related socioeconomic status measure: a cross-sectional study. BMJ Open 2016;6:e011564.

35.	Lynch BA, Finney Rutten LJ, Jacobson RM, et al. Health Care Utilization by Body Mass Index in a Pediatric Population. Acad Pediatr 2015;15:644-50.

36.	Harris MN, Lundien MC, Finnie DM, et al. Application of a novel socioeconomic measure using individual housing data in asthma research: an exploratory study. NPJ primary care respiratory medicine 2014;24:14018.

37.	Butterfield MC, Williams AR, Beebe T, et al. A two-county comparison of the HOUSES index on predicting self-rated health. J Epidemiol Community Health 2011;65.

38.	Juhn YJ, Beebe TJ, Finnie DM, et al. Development and initial testing of a new socioeconomic status measure based on housing data. J Urban Health 2011;88.

39.	Johnson MD, Urm SH, Jung JA, et al. Housing data-based socioeconomic index and risk of invasive pneumococcal disease: an exploratory study. Epidemiology and infection 2013;141:880-7.

40.	Hammer R, Capili C, Wi C-I, et al. A new socioeconomic status measure for vaccine research in children using individual housing data: a population-based case-control study. BMC Public Health 2016;16:1-9.

41.	Juhn YJ, Ryu E, Wi CI, et al. Assessing socioeconomic bias in machine learning algorithms in health care: a case study of the HOUSES index. Journal of the American Medical Informatics Association 2022;29:1142-1151.
