# Supplementary material for: Beyond biological risk: socioeconomic vulnerability and adverse pediatric kidney transplant outcomes
Source: Pediatr Nephrol. Author manuscript; Available in PMC 2026 Aug 21. (PMC13481395; doi:10.1007/s00467-026-07322-6)
Supplement: Supplementary file 2 [file NIHMS2199671-supplement-MOESM3.docx]

Supplementary Table 3: Patient characteristics by ADI

|  | **1–3 (N=121)** | **4 (N=16)** | **p value** |
| --- | --- | --- | --- |
| **Age at Transplant** |  |  | 0.680^1^ |
| Mean (SD) | 11.614 (5.105) | 12.169 (4.439) |  |
| Median (Q1, Q3) | 12.630 (7.210, 15.910) | 13.450 (8.967, 14.830) |  |
| Range | 2.060–19.610 | 2.330–18.630 |  |
| **Sex** |  |  | 0.158^2^ |
| F | 53 (43.8%) | 10 (62.5%) |  |
| M | 68 (56.2%) | 6 (37.5%) |  |
| **Race** |  |  |  |
| White | 92 (76.0%) | 11 (68.8%) |  |
| Black | 11 (9.1%) | 1 (6.2%) |  |
| Asian | 13 (10.7%) | 4 (25.0%) |  |
| American Indian or Alaska Native | 5 (4.1%) | 0 (0.0%) |  |
| Other | 0 (0.0%) | 0 (0.0%) |  |
| **HOUSES Binary Group** |  |  |  |
| 1 | 33 (27.3%) | 5 (31.2%) |  |
| 2–4 | 88 (72.7%) | 11 (68.8%) |  |
| (Missing) | 0 (0.0%) | 0 (0.0%) |  |
| **COI National Binary Group** |  |  |  |
| 1 | 18 (14.9%) | 5 (31.2%) |  |
| 2–4 | 103 (85.1%) | 11 (68.8%) |  |
| (Missing) | 0 (0.0%) | 0 (0.0%) |  |
| **COI State Binary Group** |  |  |  |
| 1 | 51 (42.1%) | 15 (93.8%) |  |
| 2–4 | 70 (57.9%) | 1 (6.2%) |  |
| (Missing) | 0 (0.0%) | 0 (0.0%) |  |
| **Binary Insurance** |  |  | 0.357^2^ |
| Private | 75 (62.0%) | 8 (50.0%) |  |
| Public/Other | 46 (38.0%) | 8 (50.0%) |  |
| **Pretransplant Dialysis** |  |  | 0.328^2^ |
| N | 37 (30.6%) | 3 (18.8%) |  |
| Y | 84 (69.4%) | 13 (81.2%) |  |
| **Time on Dialysis** |  |  | 0.778^2^ |
| Not on dialysis | 37 (30.6%) | 3 (18.8%) |  |
| <1 year | 49 (40.5%) | 8 (50.0%) |  |
| 1–3 years | 26 (21.5%) | 3 (18.8%) |  |
| >3 years | 3 (2.5%) | 1 (6.2%) |  |
| Unknown time on dialysis | 6 (5.0%) | 1 (6.2%) |  |
| **Donor Type** |  |  | 0.507^2^ |
| Deceased | 56 (46.3%) | 6 (37.5%) |  |
| Living | 65 (53.7%) | 10 (62.5%) |  |
| **HLA Mismatch** |  |  | 0.977^1^ |
| N-Miss | 3 | 1 |  |
| Mean (SD) | 3.788 (1.501) | 3.800 (1.474) |  |
| Median (Q1, Q3) | 4.000 (3.000, 5.000) | 4.000 (3.000, 5.000) |  |
| Range | 0.000–6.000 | 1.000–6.000 |  |
| **CDC BMI Z-Score** |  |  | 0.915^1^ |
| Mean (SD) | 0.030 (1.397) | 0.069 (1.104) |  |
| Median (Q1, Q3) | 0.111 (-0.798, 1.080) | -0.226 (-0.677, 1.134) |  |
| Range | -4.053–2.445 | -2.000–1.890 |  |
| **Cause of Kidney Failure** |  |  | 0.948^2^ |
| Congenital anomalies | 55 (45.5%) | 6 (37.5%) |  |
| FSGS | 21 (17.4%) | 4 (25.0%) |  |
| Glomerulonephritis | 6 (5.0%) | 1 (6.2%) |  |
| Other | 30 (24.8%) | 4 (25.0%) |  |
| Unknown | 9 (7.4%) | 1 (6.2%) |  |

^1^Linear Model ANOVA

^2^Pearson’s Chi-squared test

Supplementary Table 4: Patient Characteristics by COI (State)

|  | COI 1 (N =66) | COI 2–4 (N = 71) | P value |
| --- | --- | --- | --- |
| **Age at Transplant** |  |  | 0.906^1^ |
| Mean (SD) | 11.732 (5.134) | 11.630 (4.948) |  |
| Median (Q1, Q3) | 13.150 (7.475, 15.477) | 12.510 (7.625, 16.030) |  |
| Range | 2.060–19.610 | 2.130–18.460 |  |
| **Sex** |  |  | 0.111^2^ |
| F | 35 (53.0%) | 28 (39.4%) |  |
| M | 31 (47.0%) | 43 (60.6%) |  |
| **Race** |  |  |  |
| White | 45 (68.2%) | 58 (81.7%) |  |
| Black | 8 (12.1%) | 4 (5.6%) |  |
| Asian | 10 (15.2%) | 7 (9.9%) |  |
| American Indian or Alaska Native | 3 (4.5%) | 2 (2.8%) |  |
| Other | 0 (0.0%) | 0 (0.0%) |  |
| **HOUSES Binary Group** |  |  |  |
| 1 | 23 (34.8%) | 15 (21.1%) |  |
| 2–4 | 43 (65.2%) | 56 (78.9%) |  |
| (Missing) | 0 (0.0%) | 0 (0.0%) |  |
| **ADI Binary Group** |  |  |  |
| 1–3 | 51 (77.3%) | 70 (98.6%) |  |
| 4 | 15 (22.7%) | 1 (1.4%) |  |
| (Missing) | 0 (0.0%) | 0 (0.0%) |  |
| **COI National Binary Group** |  |  |  |
| 1 | 23 (34.8%) | 0 (0.0%) |  |
| 2–4 | 43 (65.2%) | 71 (100.0%) |  |
| (Missing) | 0 (0.0%) | 0 (0.0%) |  |
| **Binary Insurance** |  |  | 0.015^2^ |
| Private | 33 (50.0%) | 50 (70.4%) |  |
| Public/Other | 33 (50.0%) | 21 (29.6%) |  |
| **Pretransplant Dialysis** |  |  | 0.108^2^ |
| N | 15 (22.7%) | 25 (35.2%) |  |
| Y | 51 (77.3%) | 46 (64.8%) |  |
| **Time on Dialysis** |  |  | 0.122^2^ |
| Not on dialysis | 15 (22.7%) | 25 (35.2%) |  |
| <1 year | 25 (37.9%) | 32 (45.1%) |  |
| 1–3 years | 19 (28.8%) | 10 (14.1%) |  |
| >3 years | 2 (3.0%) | 2 (2.8%) |  |
| Unknown time on dialysis | 5 (7.6%) | 2 (2.8%) |  |
| **Donor Type** |  |  | 0.156^2^ |
| Deceased | 34 (51.5%) | 28 (39.4%) |  |
| Living | 32 (48.5%) | 43 (60.6%) |  |
| **HLA Mismatch** |  |  | 0.093^1^ |
| N-Miss | 2 | 2 |  |
| Mean (SD) | 4.016 (1.453) | 3.580 (1.509) |  |
| Median (Q1, Q3) | 4.000 (3.000, 5.000) | 4.000 (2.000, 5.000) |  |
| Range | 0.000 - 6.000 | 0.000 - 6.000 |  |
| **CDC BMI Z-Score** |  |  | 0.247^1^ |
| Mean (SD) | 0.175 (1.232) | -0.095 (1.470) |  |
| Median (Q1, Q3) | 0.235 (-0.753, 1.187) | 0.001 (-0.842, 0.979) |  |
| Range | -2.325 - 2.387 | -4.053 - 2.445 |  |
| **Cause of Kidney Failure** |  |  | 0.614^2^ |
| Congenital anomalies | 27 (40.9%) | 34 (47.9%) |  |
| FSGS | 11 (16.7%) | 14 (19.7%) |  |
| Glomerulonephritis | 5 (7.6%) | 2 (2.8%) |  |
| Other | 17 (25.8%) | 17 (23.9%) |  |
| Unknown | 6 (9.1%) | 4 (5.6%) |  |

^1^Linear Model ANOVA

^2^Pearson’s Chi-squared test
